# Supplementary material for: Chromothripsis during telomere crisis is independent of NHEJ, and consistent with a replicative origin
Source: Genome Res. 2019 May;29(5):737–49. doi: 10.1101/gr.240705.118 (PMC6499312; doi:10.1101/gr.240705.118)
Supplement: Supplemental Material [file supp_gr.240705.118_Supplemental_file_1.zip › contigs/annotated_contigs/DB37/contig.2.DB37_length_662_mean_cov_5.21148036254.docx]

**DB37_length_662_mean_cov_5.21148036254**

```
CCTTCTGAACAGTCATATTTGTCTCTAACTTAAACCTACTATATATC|TATATATATATATATATATATATATATATATATATATATAA
                                                >chr9:107707069-107707302 + E=2e-128 p=5
AATGTATTATTGTTTTGATTTATATTTTCTTTGATTAAACTGAAAAATAATATGGATTAAATAAAAATAAAGATTAAACATCTTTTCAT
e-02
GTTTATCGGTGTTTAAAATCCAATGTTTATTCTTTCCTTATTTATCTGTTAAAAGCTCTTGTTAAACCAAGTGTTTCAACTTTCTGACT

GTTACATGTGTT|GC|TACTTGGGAGGTTGAGGCATGAGAATTACTTGAACCTGGGAATCAGAGGTTGCACTGAGCCAAGATTGTGCCA
             >chr9:107712345-107712729 + E=2e-216
CTGCACTCCAGCCCAGGTGATGGAGCGAGACTCTGTCTCAAAAAACAAACAAACAAACAAACAAAACATTGTCTTTCTTTATATCCTTA

TTCTATGAGCTTTTTTCTATTAAAAAAATTGTTTTAACTGATTAAACTTTGTTGTTAAAAACTAATATGTTAAAAACACACATATTAGC

CTAGGCCTACACAGGGTCAGGATCATCACTATCATAGACTTCCACCTCACATCTTGTCCCACTGGAAGGTCTTCAAGGGCAGTAACATG

CATGGAGCTTTCATCTCCTATGATAACAATGCCTTTCTTCTG
```
